# Comparison of debris extrusion during retreatment using two different File systems and two irrigation activation systems

**DOI:** 10.34172/joddd.025.42344

**Published:** 2025-12-31

**Authors:** Selen İnce Yusufoglu, Beyza Turan Saglam, Feyza Nur Ergüner

**Affiliations:** Department of Endodontics, Faculty of Dentistry, Ankara Yıldırım Beyazıt University, Ankara, Turkey

**Keywords:** Debris extrusion, EasyDo activator, PUI, Reciproc, Retreatment

## Abstract

**Background.:**

This study aimed to assess the quantity of apically extruded debris generated during root canal retreatment.

**Methods.:**

Sixty single-rooted teeth were shaped and obturated with gutta-percha and a resin-based sealer. After three weeks, the samples were prepared for retreatment. They were then divided into four groups (n=15): OneShape+PUI, OneShape+EasyDoActivator, Reciproc+PUI, and Reciproc+EasyDoActivator. Apically extruded debris was gathered and placed in previously weighed Eppendorf tubes. The data were statistically analyzed.

**Results.:**

With ultrasonic irrigation, debris extrusion was noticeably higher with the OneShape system (*P*=0.0001). In the Reciproc system, significantly more debris extrusion occurred with sonic activation than with ultrasonic activation (*P*=0.0001), while no significant difference was observed between irrigation activation systems for the OneShape system (*P*=0.841). The OneShape+EasyDoActivator group showed significantly higher debris extrusion than Reciproc+PUI (*P*=0.002).

**Conclusion.:**

Given the constraints of this study, the Reciproc file system with PUI resulted in less apical debris extrusion, proving safer in terms of periapical extrusion compared to other systems.

## Introduction

 The primary objective of root canal retreatment is to facilitate comprehensive debridement, reshaping, and obturation of the root canal system. This process involves the removal of existing filling materials, accumulated debris, and microorganisms within the root canal.^1,2^ Thorough elimination of root canal filling materials is frequently challenging to accomplish. After reaching the apical foramen, it is crucial to fully eliminate any remaining debris within the root canal and disinfect the root canal. Therefore, the mechanical preparation and irrigation stages are of great importance.^3^ Various techniques and instruments, including hand files, rotary, or reciprocating file systems, have been suggested for removing the root canal filling material.^2^ Manufacturers have developed numerous systems with rotational and reciprocating movements. When compared to hand files, reciprocating file systems have been associated with a decreased frequency of apical debris extrusion during root canal retreatment. Furthermore, they demonstrate greater effectiveness in removing filling material than rotary files.^4^ Research suggests that no single technique can fully remove root canal filling materials from complex anatomical structures, such as oval and curved canals with an isthmus. Consequently, supplementary irrigation activation techniques, including sonic and ultrasonic activation, are recommended to enhance the effectiveness of filling material removal.^2^ Passive ultrasonic irrigation (PUI) uses ultrasonic activation of irrigant solutions to effectively remove debris and microorganisms.^5^ On the other hand, sonic activation uses mechanical vibration technology for root canal treatment. The Easydo Activator (EA, Easynsmile, China) uses flexible polyamide tips with three tapered designs for apical delivery of irrigants, effectively advancing irrigants into the apical third without contacting dentin.^6^

 During retreatment, endodontic instruments are used with mild to moderate pressure. This application may unintentionally displace necrotic pulp tissue, bacteria, irrigants, or obturation materials toward the apical region.^7^ Materials extruded apically are clinically implicated in flare-ups, postoperative inflammation, and compromised apical healing.^7^ The quantity of debris and filling material extruded apically is influenced by the design and kinematics of the files employed during retreatment.^8^

 The present in vitro study evaluated the volume of apically extruded debris during root canal retreatment using Reciproc and OneShape file systems in conjunction with PUI and EA irrigation activation devices. The first null hypothesis asserts that there is no difference in debris extrusion between the file systems employed, while the second null hypothesis suggests no variation in debris extrusion between the irrigation activation devices used during retreatment protocols.

## Methods

 The study received ethical approval from Ankara Yıldırım Beyazıt University’s Social and Human Sciences Ethics Committee (Approval No: 2023/161). Sixty single-rooted, caries-free teeth extracted for orthodontic or periodontal reasons were included. The teeth were inspected under × 20 magnification using a stereomicroscope (Olympus BX43; Olympus Co, Tokyo, Japan) for fractures, cracks, or defects, and those with such issues or open apices were excluded. Each tooth was assessed using buccolingual and mesiodistal radiographs, and teeth with calcification, resorption, curved canals, multiple canals, or open apices were replaced with suitable teeth. The teeth were maintained in 0.9% saline solution until they were ready for use.

 The coronal part of each tooth was sectioned below the cementoenamel junction under water cooling, resulting in root lengths of 13 ± 1 mm. The root canals were negotiated with a #15 K-file (Dentsply Maillefer, Ballaigues, Switzerland) until the file tip reached the apical foramen, upon which the working length was determined at 1 mm short of this point.

 Root canal shaping was carried out with Perfect Root files (IMD; Shanghai, China) to an apical size of 0.06, adhering to the manufacturer’s guidelines (torque setting: 4–5.2 Ncm, speed setting: 300 rpm) and using an endodontic motor (VDW, Munich, Germany). After each file change, the root canals were flushed with 2 mL of 2.5% NaOCl (Sodium Hypochlorite, Werax, Izmir, Turkey). In the final irrigation step, all the samples were rinsed with 2 mL of 17% EDTA (Ethylenediaminetetraacetic Acid, Werax, Izmir, Turkey) followed by 2 mL of distilled water, and the root canals were subsequently dried with paper points (Diadent, Diadent International). They were then obturated with gutta-percha and Adseal sealer (Metabiomed, Cheongju, Korea). Utilizing Cavit G (3M Espe, Seefeld, Germany), the access cavities were temporarily sealed. To allow the sealer to fully set, the samples were kept at 37 °C and 100% humidity for three weeks. Following this, they were divided into four retreatment subgroups.

###  Preparation of Eppendorf tubes, device setup, and irrigation procedures

 A 10^-4^ precision scale (Precisa XB 220A, Precisa Instruments, Dietikon, Switzerland) was used to weigh each empty Eppendorf tube three times. The starting weight was noted as the computed mean weight. Myers and Montgomery’s approach^9^ was then used to put the empty Eppendorf tubes into glass jars. Each jar was fitted with a stopper with a hole to accommodate each tooth. Needles were placed into the stoppers to equalize internal and external air pressure. Rubber dams were used to isolate the setup, preventing irrigant leakage and allowing operator blinding during instrumentation and irrigation procedures.

###  Experimental groups

 Group 1: OneShape file and PUI activation (n = 15). Retreatment was performed with the OneShape (25/.06) file (Micro-Mega, Besançon, France), following the manufacturer’s instructions (torque setting: 4 Ncm, speed setting: 350–450 rpm). Following each use of the file, each root canal was irrigated with 2 mL of distilled water administered through a 27-gauge syringe. After reaching the apical foramen, the root canals were activated using PUI with 2.5 mL of distilled water, employing a #25 ultrasonic tip (Woodpecker, Japan) positioned 1 mm short of the working length and operated across four power levels.

 Group 2: OneShape file and Sonic activation (n = 15). Retreatment was performed similar to that in group 1. For final irrigation activation, 2.5 mL of distilled water was used with EasyDoActivator (Easy in Smile, China), equipped with a red medium (25/04) tip located 1 mm short of the established working length and activated at 3,100 rpm.

 Group 3: Reciproc file and PUI activation (n = 15). Retreatment was carried out according to the manufacturer’s instructions using the Reciproc (25/.08) file (VDW, Munich, Germany). After each file use, the root canals were irrigated with 2 mL of distilled water using a 27-gauge syringe. Final irrigation was performed with PUI and 2.5 mL of distilled water, activating with a #25 ultrasonic tip 1 mm short of working length.

 Group 4: Reciproc file and sonic activation (n = 15). Retreatment was performed as in group 3. Final irrigation activation used EasyDoActivator and a red medium (25/04) tip, activated at 3,100 rpm.

 To enhance measurement accuracy, distilled water was used during both instrumentation and final irrigation. This approach prevents potential crystallization of sodium hypochlorite or other irrigants, which could otherwise affect debris extrusion results.

 For five days, the Eppendorf tubes were kept in an incubator at 70°C. Once the irrigants had fully evaporated, each tube was weighed three times. The mean weight was subsequently recorded as the final weight. The weight of the extruded debris was determined by subtracting the initial weight from the final weight.

###  Statistical analysis

 Normality of the data was evaluated using the Shapiro-Wilk test, followed by statistical analysis with one-way ANOVA and t-tests. SPSS 21.0 (IBM-SPSS Inc., Chicago, USA) was used for all analyses, and a significance threshold of 5% was used.

## Results

Table 1 displays the mean and standard deviation values for each group. All the file systems caused apical debris extrusion, according to the results, with significant differences between the groups (*P* = 0.0001, *P* < 0.05). The Reciproc + PUI group showed a significantly lower amount of debris extrusion (*P* = 0.002). Although the use of EA activation with both the OneShape and Reciproc file systems resulted in higher debris extrusion, the differences between these groups were not significant (*P* > 0.05). When PUI irrigation was used, the OneShape file system resulted in a significantly greater amount of debris extrusion (*P* = 0.0001). For the Reciproc file system, EA activation led to more debris extrusion compared to the ultrasonic technique (*P* = 0.0001). In contrast, no significant difference was observed between the irrigation activation methods when using the OneShape file system (*P* = 0.841).

**Table 1:** The mean and standard deviation values

**Groups**	**Mean±Std**
Oneshape + PUI	0.0139 ± 0.0007^a^
Oneshape + EA	0.0142 ± 0.0013^a^
Reciproc + PUI	0.0086 ± 0.0008^b^
Reciproc + EA	0.0143 ± 0.001^a^

PUI: Passive ultrasonic irrigation; EA: Easydo Activator. ^a,b^Means with the same letter are not significantly different from each other.

## Discussion

 Residual obturating material within the root canal adversely affects the success of retreatment procedures. Selecting the most efficient and rapid technique during retreatment is essential for clinicians. The volume of apically extruded debris is clinically significant, as it is associated with periapical inflammation, flare-ups, postoperative pain, and impaired healing of periapical tissues. This study evaluated the degree of apical debris extrusion during retreatment procedures using two distinct file systems and irrigation activation devices. Observing significant differences in apical debris extrusion between the two file systems and the two activation devices led to the rejection of the null hypotheses H0 and H1.

 In the present study, the type of irrigation activation method significantly influenced debris extrusion when combined with different instrumentation systems. The Reciproc + PUI group demonstrated the lowest level of debris extrusion, whereas EndoActivator (EA) activation tended to produce higher extrusion values, particularly when used with the Reciproc system. These findings are consistent with previous studies reporting that PUI is generally more effective at controlling apical extrusion than sonic activation systems, such as the EA. Sümbüllü et al^10^ observed that EA and EDDY produced significantly greater apical debris extrusion than PUI, manual dynamic agitation, and needle irrigation. Similarly, Haupt et al^11^ found that PUI was more efficient at removing debris and the smear layer from the apical third than sonic activation, suggesting superior cleaning efficacy with less apical extrusion risk.Ada et al^12^ and İnce Yusufoglu et al^13^ reported that PUI resulted in less apical debris extrusion than sonic activation. The superior performance of PUI may be attributed to its acoustic streaming and cavitation effects, which enhance irrigant penetration and debris suspension without creating excessive apical pressure.^5^ In contrast, the sonic motion generated by the EndoActivator produces lower-frequency oscillations and limited acoustic streaming, which may result in less effective debris removal from the apical region and, under certain conditions, greater apical extrusion.^14^ In the current study, when the Reciproc file system was combined with EA activation, debris extrusion increased significantly compared with ultrasonic activation. This observation may be explained by the file’s reciprocating motion, which tends to accumulate debris in the apical region; subsequent sonic agitation could displace this material apically. On the other hand, when using the OneShape rotary system, no significant difference was found between EA and PUI activation, possibly due to the continuous rotation producing a more even debris distribution within the root canal and less accumulation at the apex before activation.

 The Reciproc file system is a single-file system characterized by an S-shaped design and is produced using M-wire technology. The results of earlier studies on apical debris extrusion linked to the Reciproc file system during primary root canal therapy have been mixed.^15,16^ Koçak et al^17^ and De-Deus et al^16^ suggest that the Reciproc file system extrudes less debris apically. In contrast, Bürklein et al^15^ argue that Reciproc files extrude more debris than OneShape files. In retreatment studies, according to Lu et al,^18^ the Reciproc file system resulted in more debris extrusion than the MTwo system. In contrast, studies by Dincer et al^4^ and Uzunoğlu and Aktemur Türker^19^ demonstrated that the Reciproc file system extruded less debris than other file systems. Another study reported that the Reciproc file system results in less debris extrusion than ProTaper Universal and R Endo files.^20^ Our findings are consistent with these studies, demonstrating that Reciproc combined with PUI led to a markedly reduced amount of apical debris extrusion in comparison to the other groups. These differences may stem from kinematic, cross-sectional, and taper variations among file systems. Previous studies also show that instrumentation techniques and file designs affect apical debris extrusion.^19,21^

 This study selected mandibular premolars based on their type, canal configurations, working length, and degree of curvature. Single-rooted teeth were selected because of the practicality and simplicity of standardization. Other studies have also used single-rooted teeth with minimal curvatures.^22-24^ On the other hand, results may vary if multi-rooted teeth with severe curvatures are used.

 To prevent crystallization, distilled water was used instead of sodium hypochlorite, which could affect dentin weight and undermine the reliability of the findings.^25^ The use of distilled water eliminates the risk of crystallization or residue formation that can occur when sodium hypochlorite (NaOCl) or other chemical irrigants evaporate or react with dentin particles. Such crystallization can artificially increase the dry weight of extruded material, thereby overestimating the amount of debris extruded.^25^ Several authors have emphasized that when the aim is to quantify debris by weight, chemically inert solutions such as distilled water or saline should be preferred to avoid confounding factors such as crystallized irrigant residues.^15,26^ Although distilled water does not replicate the clinical antimicrobial environment of NaOCl, its use in in vitro models is widely accepted to achieve standardized and reproducible debris quantification.^27^ Apically extruded debris was collected using the well-known Myers-Montgomery technique. The main limitation of this method is its inability to simulate periapical tissue, as the absence of periapical backpressure did not prevent apical extrusion. This represents a key limitation of our study. Another limitation of this study is that the instruments were compared using their original tapers, which may have influenced the amount of apical debris extrusion. Each file system is designed with a fixed taper and a specific geometric configuration that reflects its intended cutting efficiency, debris-removal capability, and shaping characteristics. To preserve clinical relevance and standardization within each system, the instruments were used according to the manufacturer’s instructions rather than being artificially equalized. Using files with identical taper values would have required modifications or non-standard instruments, which could compromise the validity of the comparison by failing to reflect their actual clinical performance. Standardizing the taper across all systems could have allowed a more isolated evaluation of the effects of file kinematics and design.

## Conclusion

 Given the limitations of this study, retreatment procedures using the Reciproc file system combined with PUI were found to cause less apical debris extrusion, suggesting this option is more favorable.

## Competing Interests

 The authors declare no competing interests with regard to the authorship and/or publication of this article.

## Ethical Approval

 This study was approved by the Institutional Review Board (reference no. 2023-161).
